# A New Self-Sensing Fiber Optic Anchor to Monitor Bolt Axial Force and Identify Loose Zones in the Surrounding Rock of Open TBM Tunnels

**DOI:** 10.3390/s24206709

**Published:** 2024-10-18

**Authors:** Xin Kang, Xiongyao Xie, Kun Zeng

**Affiliations:** 1 Department of Geotechnical Engineering, College of Civil Engineering, Tongji University, Shanghai 200092, China; kxcumtb@163.com (X.K.); zengkun_tj@tongji.edu.cn (K.Z.); 2 State Key Laboratory of Disaster Reduction in Civil Engineering, Tongji University, Shanghai 200092, China; 3 Key Laboratory of Geotechnical and Underground Engineering of Ministry of Education, Tongji University, Shanghai 200092, China

**Keywords:** bolt axial force, loose zone, self-sensing optical fiber anchor, borehole inspection

## Abstract

TBM has been widely used in underground engineering and construction, but there is no precedent for the application of open TBM in the inclined shafts of coal mines, which brings new challenges to the support system. The distribution of the axial forces on anchors and the range of loosening of the surrounding rock are crucial considerations in tunnel support design. Existing methods for measuring the axial forces in anchors and determining the extent of loosening in the surrounding rock typically remain at the inspection level, lacking long-term and real-time monitoring capabilities. This paper presents a new self-sensing anchor with embedded optical fibers (made using an improved stirrer) and proposes an intelligent tunnel rock monitoring system. The paper also outlines a method for identifying loosening zones in surrounding rock based on monitoring data and theoretical analysis. Installing self-sensing anchors in the deep sections of the rock surrounding a tunnel provides three-dimensional, round-the-clock real-time monitoring of the axial forces acting on the anchors, using new technology and methods to recognize the deformation characteristics of loosening zones within the surrounding rock. This new self-sensing fiber optic anchor was first applied to an open TBM tunneling project in an inclined shaft in the Kekegai coal mine, and monitoring data indicate that self-sensing optical fiber anchors can accurately reflect stress patterns in real time. The axial force curve can be divided into four segments: the borehole area, the loosening zone, the stable zone, and the anchoring zone. Consequently, it accurately identifies the thickness of loosening zones at different positions within the tunnel’s surrounding rock. This information is compared and verified against results obtained from bolt dynamometers and borehole inspection. On this basis, an intelligent monitoring system was established to provide a basis for making engineering construction decisions, which makes tunnel construction smarter and helps technicians timely adjust TBM driving and support parameters.

## 1. Introduction

Although open TBM has been widely used in underground engineering construction, there is no precedent for its application in the inclined shafts of coal mines. Compared with traditional tunneling technology, TBM has a fast tunneling speed, a high degree of mechanization, and requires fewer people to operate, but it brings new challenges to creating a support system during rapid construction.

Anchors, as commonly used support materials in underground construction, play a crucial role in controlling the surrounding rock. Knowing their operational status is essential to analyzing the effectiveness of support systems. However, due to the fact that anchors need to be anchored inside the surrounding rock, their installation locations are often concealed, making it challenging to proactively detect issues during the actual construction process. Problems with anchors bending or breaking are usually discovered only after incidents or accidents occur, leading to a reactive approach to a problem that is difficult to address proactively.

Therefore, full-length and real-time monitoring of the distribution of axial forces on and in an anchor is of paramount importance to understanding the stability of the surrounding rock and the safety of tunnels. This proactive approach is crucial to preventing potential issues.

Traditional methods of monitoring anchor stress include resistance strain gauges, vibrating wire sensors, and point sensors such as fiber Bragg grating sensors. These methods can monitor specific points or a few points on the anchor, but they may not provide full coverage.

In recent years, fiber optic sensing technology has emerged as a monitoring technique. Due to the small size of optical fibers, their strong resistance to interference, and the relatively high accuracy of their measurement results, they have gained popularity in underground engineering deformation monitoring. Fiber optic sensors can achieve distributed, long-term, and real-time automated monitoring, making them a research hotspot in the field of underground engineering deformation monitoring [1,2].

Buchmayer et al. [3] presented a tunnel monitoring approach based on distributed fiber optic sensing (DFOS), which monitors hundreds of strain and temperature sensing points inside the structure and gives completely new information about the behavior of the tunnel lining.

Some scholars have also used optical fibers to monitor structural damage and deformation. For example, Takeda et al. [4] proposed fiber optic-based damage monitoring of carbon-fiber reinforced plastic (CFRP) bolted joints. Monsberger et al. [5,6] installed the distributed fiber optic sensing system in the shotcrete lining in the core of a fault zone in a railway tunnel under construction.

We can refer to the above content to study the technology of optical fiber used to monitor the axial force on anchor rods and the loose zones of surrounding rock.

Chai et al. [7,8] and Liang et al. [9] have designed a novel anchor stress monitoring system using optical fibers. This system determines the stress and strain at different measurement points by analyzing the changes in reflected wavelengths and assesses the overall stress distribution. They compared the measurement accuracy of this system with traditional strain gauges. Li et al. [10] and Gao et al. [11] also conducted research on the use of fiber Bragg gratings and distributed fiber optics to monitor the axial forces on anchor bodies. However, the design of their fiber optic bolt was limited, and the axial force on the full length of the bolt body could not be monitored.

You et al. [12,13] proposed an equivalent estimation method (EEM) for a quasi-distributed deflection estimation using onboard strain data. Experimental validation tests were performed, and they demonstrated the excellent quasi-distributed sensing capability of the EEM.

Feng [14] analyzed the stress characteristics of fully anchored anchors and then conducted a comprehensive analysis of the effectiveness of a fiber optic load-sensing anchor through similarity simulation experiments. However, the design of the fiber optic bolt used is complex and cannot meet the requirements of rapid construction on site.

The theory of loosening zones has become an essential component of underground engineering construction and support structure design. Monitoring the extent and characteristics of loosening zones during construction and operation is of significant importance for tunnel safety. There are various methods for monitoring loosening zones, including multi-point displacement sensors, acoustic methods, and geological radar technology [15,16].

Gong et al. [17] used both acoustic and multi-point displacement sensor methods to test the extent of surrounding rock’s loosening zones. Gao et al. [18] and Xu et al. [19] used geological radar to detect the loosening zones of surrounding rock. They initially determined the radar frequency and time window based on field data. Yue et al. [20] believed that the dielectric constant of the surrounding rock could change due to the development of cracks in the loosening zone. The choice of radar antenna frequency needed to consider the characteristics of the surrounding rock. Zhang et al. [21] used a combination of borehole inspection instruments and geological radar to detect and analyze the extent of loosening and damage in the surrounding rock. However, these technologies are not only cumbersome, affecting the construction schedule, but also make it difficult to maintain real-time monitoring during the entire construction period.

The above research creates a new application of optical fiber to the monitoring of anchor axial force, which has a positive effect on the analysis of the stress on anchor rods and the control effect of surrounding rock. However, due to the complex construction process caused by its design defects, it cannot meet the needs of rapid excavation in construction, and it is difficult to apply to large-scale projects due to its high cost. In addition, there are few reported research studies on the identification of loose zones in surrounding rock using optical fiber.

In this paper, a new self-sensing fiber optic bolt that is cheap and can be installed quickly will be developed by improving the existing stirrer. The new bolt can be applied on a large scale in the field, so as to realize the all-round real-time monitoring of the axial force on the bolt and the loose zone of the surrounding rock. An intelligent monitoring system based on the monitoring data provides a basis for support optimization.

## 2. Designing the New Self-Sensing Optical Fiber Anchor

### 2.1. Principle of Self-Sensing Optical Fiber Anchor

**Figure 1:**
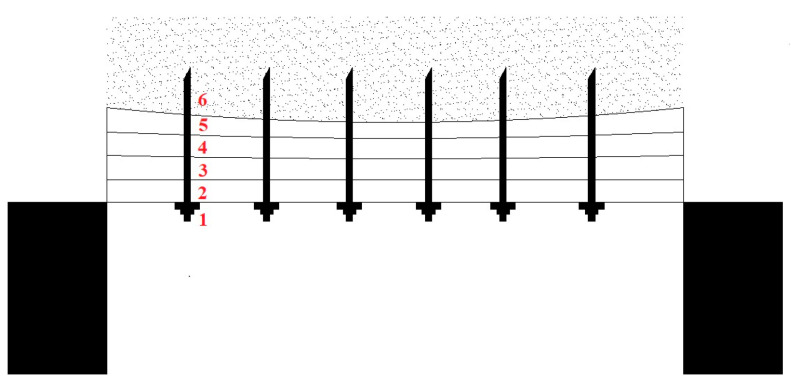
The function of a composite beam supported by bolts.

For stratified rock, the bolt will act as a composite beam, as shown in Figure 1. The axial force of the bolt can not only restrain the separation of rock layers, but also increase the friction between the rock layers to prevent them from moving. The axial force on each position of the bolt body is an important basis for reflecting the internal support’s effect on the surrounding rock.

At present, the conventional method of monitoring bolt axial force is to install a dynamometer between the nut and the tray, but this method can only monitor the axial force at point 1. The axial force at points 2–6 cannot be measured, even though the axial force of the anchor rod inside the surrounding rock is an important basis for judging the control effect of surrounding rock.

Distributed optical fiber sensing technology can obtain the axial strain distribution of an anchor rod. The axial force distribution curve of the anchor rod can be obtained by calculating the strain value at each point along the anchor rod’s axis, and the average shear stress along the anchor rod axis can be obtained from the strain value of the adjacent two points:

(1) $$\sigma_{i}=E\varepsilon_{i}$$

(2) $$\tau_{i}=\;\frac{{(\varepsilon }_{i+1}-\varepsilon_{i})EA_{b}}{\pi D∆l}$$

(3) $$N_{i}=A_{b}E\varepsilon_{i}$$

where, $\sigma_{i}$ is the stress value of the bolt’s *i* point, MPa; $\tau_{i}$ is the average shear stress between point *i* and point *i* + 1, MPa; $N_{i}$ is the axial stress at point I, KN; *D* is the anchor diameter, mm; Δ*l* is the distance between optical fiber sampling points; *E* is the elastic modulus of the anchor, MPa; and $\varepsilon_{i}$ is the strain value at the *i* point of the optical fiber, µε.

### 2.2. Manufacturing Self-Sensing Optical Fiber Anchors

#### 2.2.1. Groove

**Figure 2:**
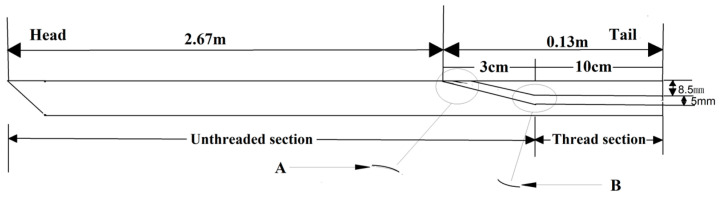
Design diagram of fiber optic anchor.

In the actual working process, besides being subjected to axial tension, the anchor also experiences significant shear stresses on its surface. To prevent the optical fiber from being affected by the surface stresses on the anchor, it needs to be arranged inside the bolt so that the optical fiber deforms synchronously with the bolt. As shown in Figure 2, taking a 22 mm × 2.8 m anchor as an example, a groove with a width × depth of 1 × 1 mm is carved into the middle surface of the 2.67 m-long bolt to accommodate the optical fiber. Through-holes measuring 4 × 4 mm are drilled in the 0.13 m-long tail of bolt to connect with the surface groove on the bolt. To prevent the optical fiber from being compressed or broken, the areas marked as A and B are designed as arcs.

#### 2.2.2. Processing

**Figure 3:**
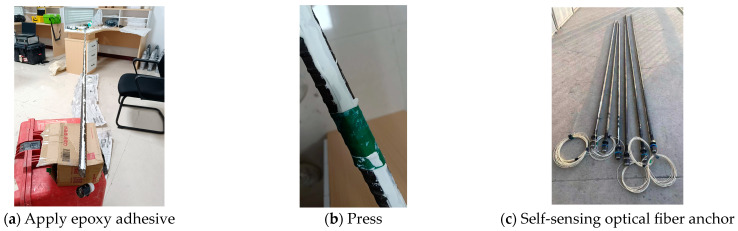
Fiber optic encapsulation.

First, sand the inside of the groove on the anchor with sandpaper and clean the groove’s surface with alcohol swabs. Then, arrange the sensors along the axial direction of the anchor. Straighten the optical fiber and place it flat against one side of the groove. In the widened section of the groove, insert the protective sheath, then fix it with epoxy adhesive, making it flush with the groove’s surface. Use PTFE compression pads and apply pressure. Next, wrap it with electrical tape and let it sit for 24 h. After the epoxy adhesive has solidified, remove the tape and PTFE compression pads, and use scissors to trim the excess optical fiber. The processing procedure is shown in Figure 3.

## 3. Engineering Application

### 3.1. Project

**Figure 4:**
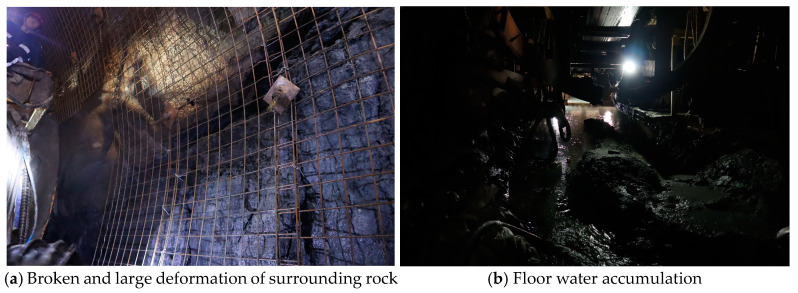
Kekegai coal mine site conditions.

The Kekegai coal mine is located in the northern area of the Yuheng Coalfield in the Jurassic coalfield of northern Shaanxi, as part of the national plan. The designed production capacity of the mine is 10.00 million tons per annum (Mt/a), with a service life of 93.0 years. The mine will be developed using inclined shafts, with the main and auxiliary shafts concentrated in the western industrial area. The main and auxiliary shaft projects are planned to be constructed using the open TBM construction method. The surrounding strata consist of typical western formations characterized by water-rich and weakly cemented rock formations. During on-site construction, the surrounding rock is severely fractured, and the support is insufficient, especially in the Luoho Formation. In this section, there is a significant amount of water in the goaf area, and the roof near the excavation face is severely fractured. This is a typical section of water-rich and weakly cemented sandstone, as shown in Figure 4.

### 3.2. Monitoring Plan

**Figure 5:**
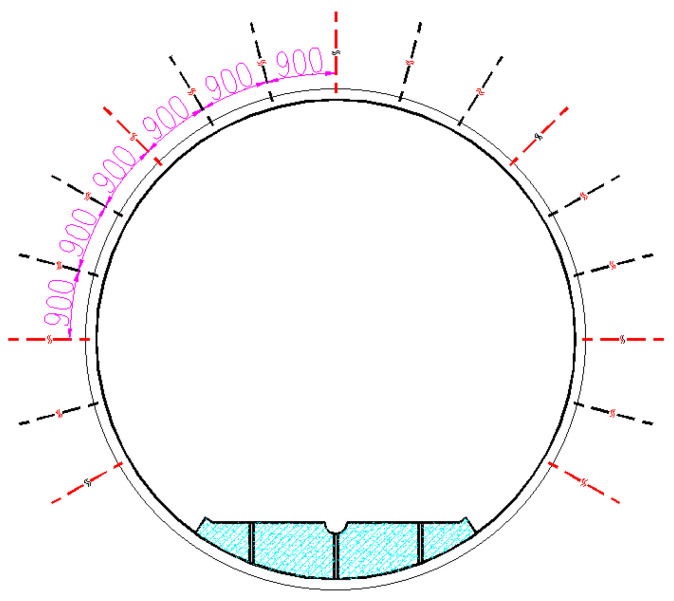
The installation positions of the self-sensing fiber optic anchor.

The selected monitoring section is a distance of 2050 m and 2100 m from the wellhead, and the specific arrangement is as shown in Figure 5. The red lines indicate the installation positions of the self-sensing fiber optic anchors, covering the top, shoulder, flank, and bottom sections. This configuration allows for a comprehensive reflection of the stress on the anchors at different locations.

### 3.3. On-Site Installation

#### 3.3.1. Installation

**Figure 6:**
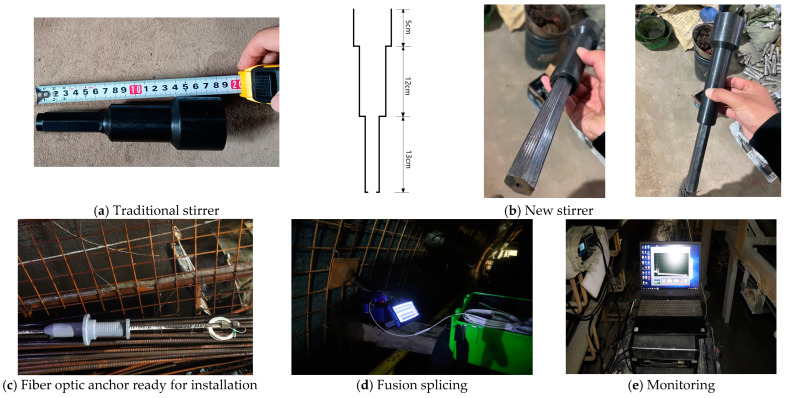
Installation of fiber optic anchor.

We followed this testing plan to install the intelligent fiber optic anchor. Before installation, perform a visual inspection of the instruments and equipment used for testing, including checking whether the optical fiber signal output is normal, among other factors. The installation process is shown in Figure 6.

The optical fiber fixed in the anchor will be damaged by the drilling machine during anchor installation. Therefore, it is necessary to ensure that the length of the exposed optical fiber after anchor installation is sufficient for fusion splicing. However, the existing stirrer has a length of 18 cm, as shown in Figure 6a, which does not meet the length requirement for exposure. A new type of stirrer needed to be designed, as shown in Figure 6b. Our new stirrer has a total length of 30 cm and a hole in the middle. Before stirring, the optical fiber is passed through it and then placed on the drilling machine. The installed fiber optic anchor is shown in Figure 6c.

#### 3.3.2. Fusion Splicing

After the intelligent anchor is installed, use a fusion splicer to splice the optical fiber reserved at the tail of the anchor with the prepared optical fiber for subsequent monitoring. The fusion splicing process is shown in Figure 6d.

#### 3.3.3. Monitoring

Use an optical fiber demodulator to collect data from the intelligent anchor, as shown in Figure 6e. This should be done immediately after installation. It serves two purposes: to check whether the installation process has damaged the monitoring equipment and to collect initial data. The measured strain values are recorded as initial values. Subsequent strain values are obtained by subtracting the initial values. This process provides the results of the optical fiber strain monitoring. The monitoring frequency is once a day. Before each reading, wipe the joints with alcohol to keep them clean. The data is also uploaded to the monitoring system in real time.

### 3.4. Anchor Bolt Dynamometer Monitoring

**Figure 7:**
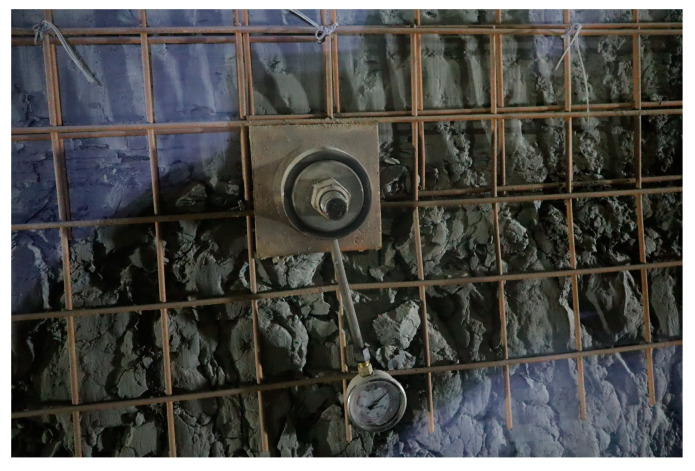
Installation of the anchor bolt dynamometer.

Due to the high research and testing costs of fiber optic anchors and their limited application scale, traditional bolt dynamometers were used to monitor the axial force on the anchors at the installation positions of the fiber optic anchors. The monitoring results were then compared with the results obtained from the fiber optic anchor monitoring. The sensor used was the MYZ-20 dial-type hydraulic bolt dynamometer, as shown in Figure 7.

### 3.5. Drilling Borehole Inspection

To validate the accuracy of identifying the loose rock zone range using self-sensing fiber optic anchors, borehole inspections were conducted on the surrounding rock in the monitoring section. This technique uses digital imaging technology to visually reflect the conditions of the rock mass in the form of videos and images. It is commonly used in coal mines to observe the development of coal seam fractures, surrounding rock fragmentation, loose zone range, and orientation. It plays an important role in analyzing the internal conditions of the surrounding rock after rock excavation.

**Figure 8:**
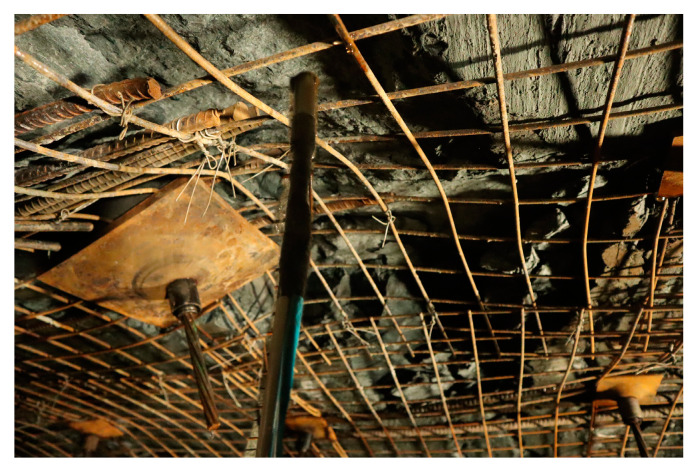
Borehole Inspection.

Borehole inspections were conducted at the top and both sides of the two monitoring sections, totaling three positions. The inspections were performed two months after support installation. The boreholes had a depth of 3 m and a diameter of 28 mm, which could accommodate a probe with a diameter of 24 mm, as shown in Figure 8.

## 4. Monitoring Result Analysis

### 4.1. Analysis of Self-Sensing Fiber Optic Anchor Monitoring Results

**Figure 9:**
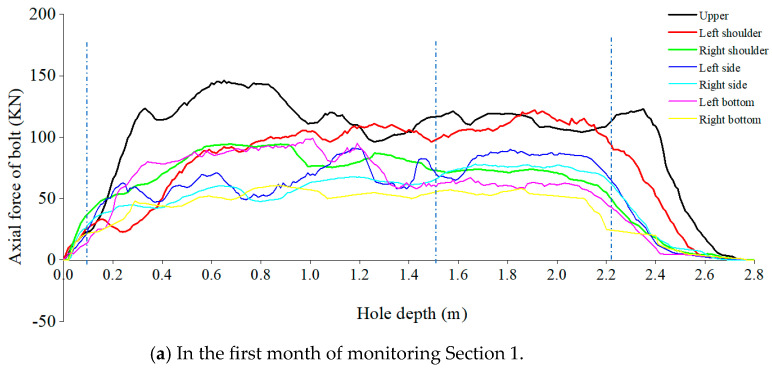

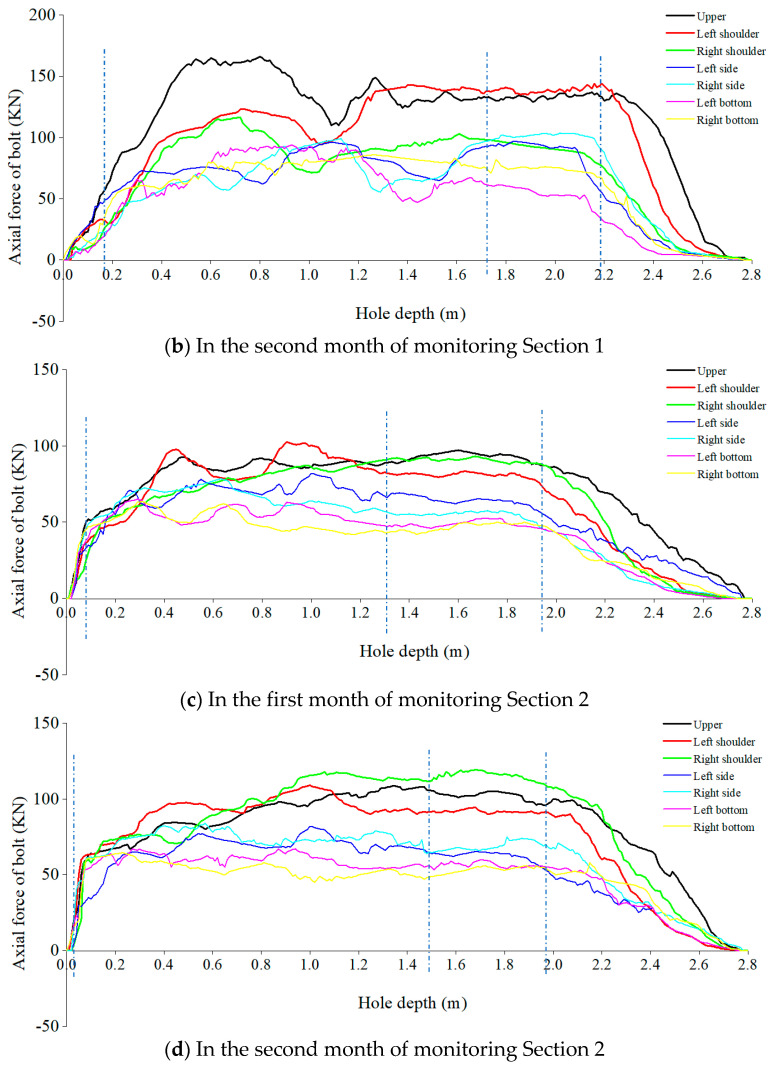
The monitoring results of the self-sensing optical fiber anchor.

After completing the support, data collection of the axial force on the fiber optic anchor was conducted. The cumulative monitoring time was 2 months, and the monitoring results are shown in Figure 9a–d. The results represent the axial forces on the fiber optic anchor at different positions in the first and second months of monitoring in Monitoring Section 1 and Monitoring Section 2.

By comparing the data from the two monitoring sections, it can be observed that in comparison to Monitoring Section 2, Monitoring Section 1 exhibits higher overall anchor axial forces with larger fluctuations. The axial forces on the anchor are not stable from the end to the tail, especially near the borehole openings. This indicates that the surrounding rock in Monitoring Section 1 is more fragmented than that in Monitoring Section 2, which aligns with the actual conditions.

The data collected during the first month for both monitoring sections show significant variations, with noticeable fluctuations in adjacent measurement points. This suggests that one month after support, the deformation of the surrounding rock has not yet stabilized, and the axial forces on the anchor continue to change in response to the deformation of the surrounding rock. In the second month, the data tend to stabilize, with measurements at different points becoming closer and the curves smoother. This indicates that at this point, the deformation of the surrounding rock has essentially concluded, and the axial forces on the anchor have reached a stable state.

Although the axial force distribution curves for each monitoring point exhibit variations, the overall trend in magnitude remains evident. From largest to smallest, the order is top > shoulder > side > bottom. However, there are slight differences in positions at different depths. For example, in Monitoring Section 1, one month after support, the axial force at the left side position in the depth range of 0.32 m to 0.98 m is second only to the arch crown force but greater than the arch waist and arch foot forces. However, at deeper positions, it is lower than the arch shoulder force.

In addition, the overall trend of the axial force curve shows a rapid increase, significant fluctuations, gradual smoothing, and slow decrease. Particularly, there is a dramatic change in axial force near the borehole opening, while the axial force near the anchored segment remains relatively stable. This indicates that the deformation of the surrounding rock becomes more intense as it gets closer to the rock surface. As the depth increases, the influence of the excavation on the surrounding rock gradually weakens and the stress approaches its original state. Based on this, it can be divided into four parts: the borehole zone, loose zone, stable zone, and anchored zone.

The borehole zone is located near the borehole opening, with a length of approximately 10 cm. Due to the exposed section where the anchor is not in a tensioned state, the axial force of the anchor increases rapidly from 0 as it enters the borehole from the exposed section. It reaches a maximum of over 60 KN, mainly at the top or shoulder position.

Moving from the borehole zone into the loose zone, due to the influence of the crushed surrounding rock squeezing deformation, the anchor experiences tension and shear stress from the surrounding rock, resulting in the most dramatic changes in axial force in this area. This indicates that this area is located within the loose zone of the surrounding rock, and this range can be used to represent the size of the loosening zone. From the data provided, it can be observed that the size of the loosening zone varies from 1 m to 1.7 m at different positions.

For example, in the case of Section 1, after one month of support, the loosening zone sizes for various positions were as follows: top (1.65 m), left shoulder (1.45 m), right shoulder (1.48 m), left side (1.6 m), right side (1.52 m), left bottom (1.33 m), and right bottom (1.1 m). After two months of support, the loosening zone sizes for the same positions were: top (1.7 m), left shoulder (1.51 m), right shoulder (1.62 m), left side (1.63 m), right side (1.6 m), left bottom (1.59 m), and right bottom (1.15 m). The ranking of loosening zone sizes from largest to smallest is: top > left side > right shoulder > right side > left shoulder > left bottom > right bottom.

In the case of Section 2, after one month of support, the loosening zone sizes for various positions were as follows: top (1.22 m), left shoulder (1.11 m), right shoulder (1.328 m), left side (1.3 m), right side (1.19 m), left bottom (1.05 m), and right bottom (0.9 m). After two months of support, the loosening zone sizes for the same positions were: top (1.33 m), left shoulder (1.25 m), right shoulder (1.45 m), left side (1.33 m), right side (1.32 m), left bottom (1.1 m), and right bottom (1.0 m). The ranking of loosening zone sizes from largest to smallest is: right shoulder > top > left side > right side > left shoulder > left bottom > right bottom.

**Figure 10:**
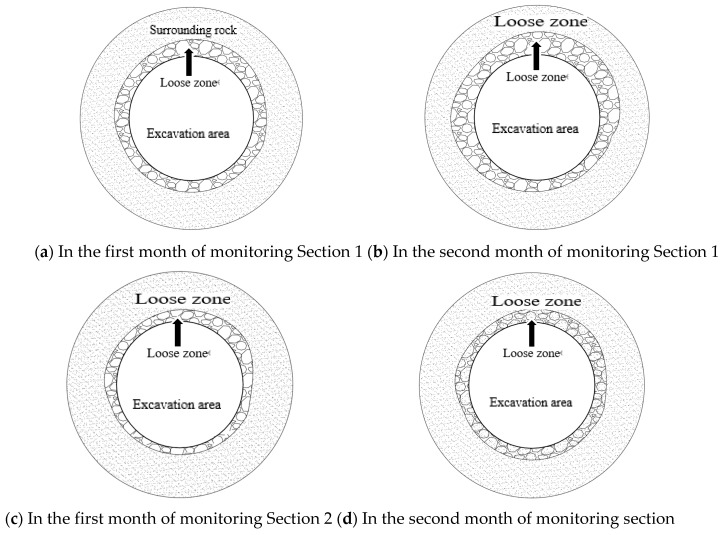
Range of loosening zone.

The loosening zone of surrounding rock as shown in Figure 10. The size of the loosening zone around the excavation chamber varied near or relative to the position and exhibited some randomness, which is caused by the stress state of the surrounding rock, the heterogeneity of the rock mass, and its anisotropy. Additionally, during the second month, the changes in axial force in the loosening zone were more intense than in the first month. This indicates that conventional support methods have a limited effect on surrounding rock control in water-rich and soft strata, and the loosening zone may continue to expand. Therefore, it is necessary to adjust the support techniques and parameters.

The stability zone, represented by the third segment of the axial force curve, showed relatively minor changes, indicating that it was within the elastic range. The surrounding rock had good integrity, and the anchor was primarily under tension with minimal shear forces. Therefore, this zone was considered stable. However, its length was relatively small, and during the second month, it became even shorter. For example, in monitoring Section 1, during the first month, the stability zone at various positions ranged from 0.4 m to 1.1 m and was located at the top and right bottom, respectively. During the second month, the stability zone at various positions ranged from 0.3 m to 1 m and was located at the left shoulder and right bottom, respectively. In monitoring Section 2, during the first month, the stability zone at various positions ranged from 0.6 m to 1.5 m and was located at the top and right bottom, respectively. During the second month, the stability zone at various positions ranges from 0.4 m to 1.2 m and is located at the right shoulder and right bottom, respectively.

The anchoring zone is characterized by a rapid decrease in axial force, with lower forces closer to the end. This indicates that this area experiences shear forces due to the action of the chemical anchor, and as it gets closer to the hole bottom, the shear forces gradually decrease. Consequently, the anchoring force acting on the anchor becomes smaller in this region.

### 4.2. Comparison of Axial Forces of Bolt

**Figure 11:**
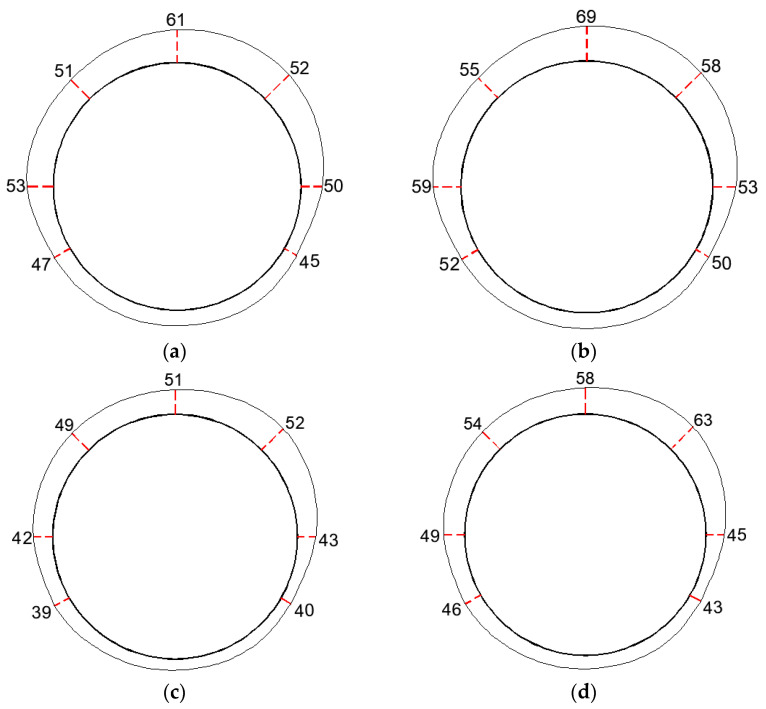
Monitoring results of anchor bolt dynamometer (KN). (**a**) In the first month of monitoring Section 1. (**b**) In the second month of monitoring Section 1. (**c**) In the second month of monitoring Section 1 In the first month of monitoring Section 2. (**d**) In the second month of monitoring Section 1 In the second month of monitoring Section 2.

The monitoring results from the bolt dynamometers for both the first and second months are shown in Figure 11. The values are given in KN. From the figure, it can be observed that in the first month after support, the maximum support force for Section 1 was 61 KN, located at the top, while the minimum was 45 KN, located at the right bottom. In the second month after support, the maximum and minimum support forces for Section 1 were 69 KN and 50 KN, respectively, still located at the top and right bottom. For Section 2, in the first month after support, the maximum and minimum support forces were 52 KN and 39 KN, respectively, located at the right shoulder and left bottom. In the second month after support, the maximum and minimum support forces for Section 2 were 63 KN and 43 KN, respectively, located at the right shoulder and right bottom.

The results from the bolt dynamometers are in close agreement with those obtained from the self-sensing optical fiber anchor in the vicinity of the anchor hole, indicating the accuracy and superiority of the self-sensing optical fiber anchor in monitoring axial forces within the surrounding rock.

### 4.3. Comparison of Loose Zone of Surrounding Rock

**Figure 12:**
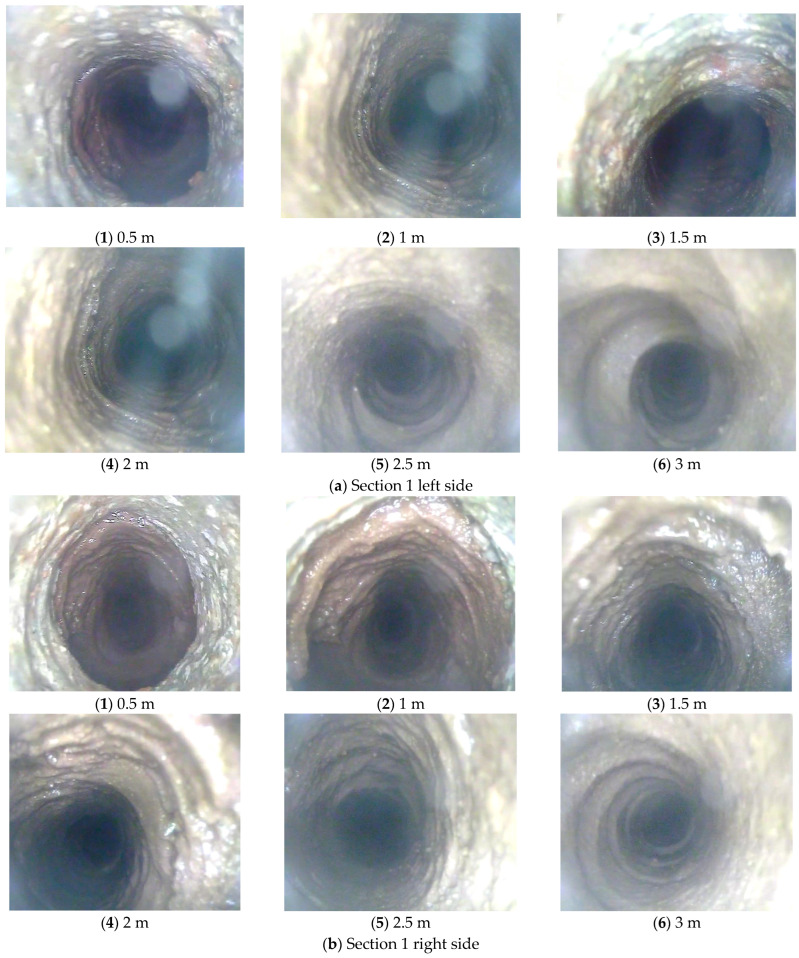

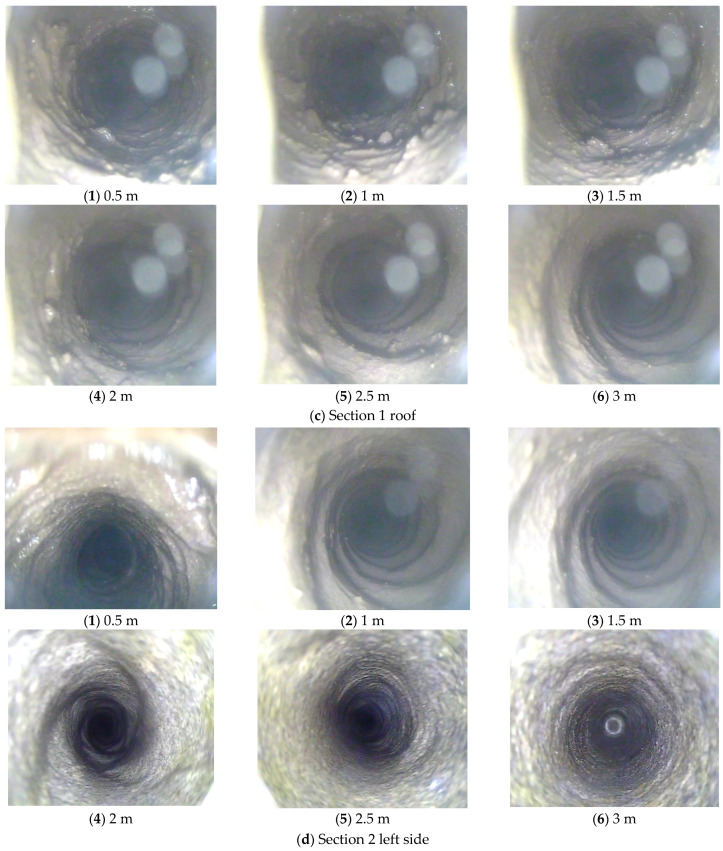

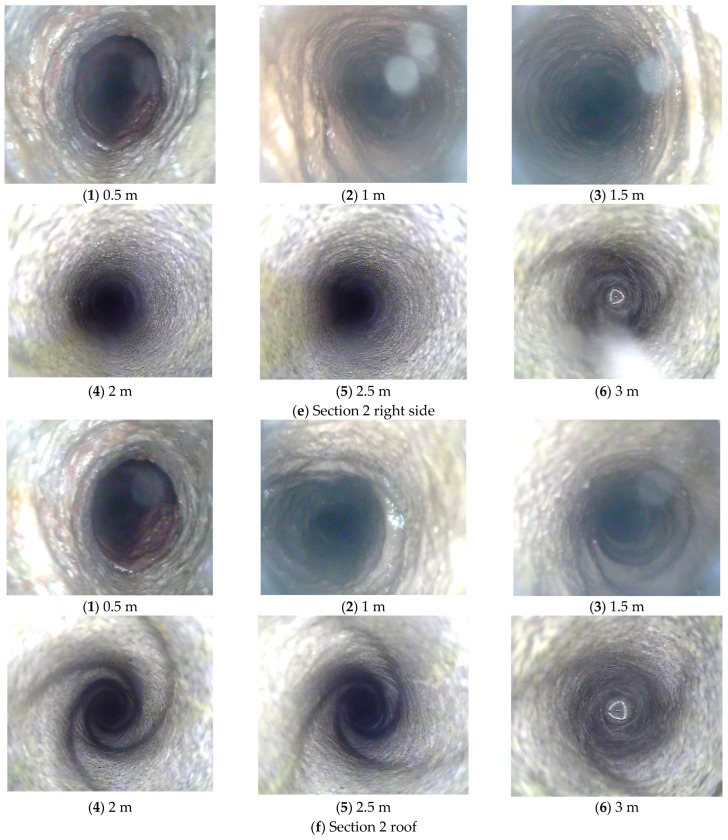
Drilling peeking video screenshot comparison.

In Figure 12, video screenshots from each borehole at 0.5 m intervals are analyzed. The specific data are shown in the figure. From the figure, it is evident that the video images from the boreholes in Section 2 are significantly clearer than those in Section 1. Especially in the deeper sections, you can clearly see the marks left by the rotating drill bit. This suggests that the surrounding rock has a higher strength and experienced less deformation. On the other hand, the video images from the boreholes in Section 1 do not show clear drill bit rotation marks from the borehole entrance to the bottom. Moreover, there is a higher water content inside the boreholes, which obstructs the lens. This indicates that the surrounding rock is fractured and damaged in this area.

**Figure 13:**
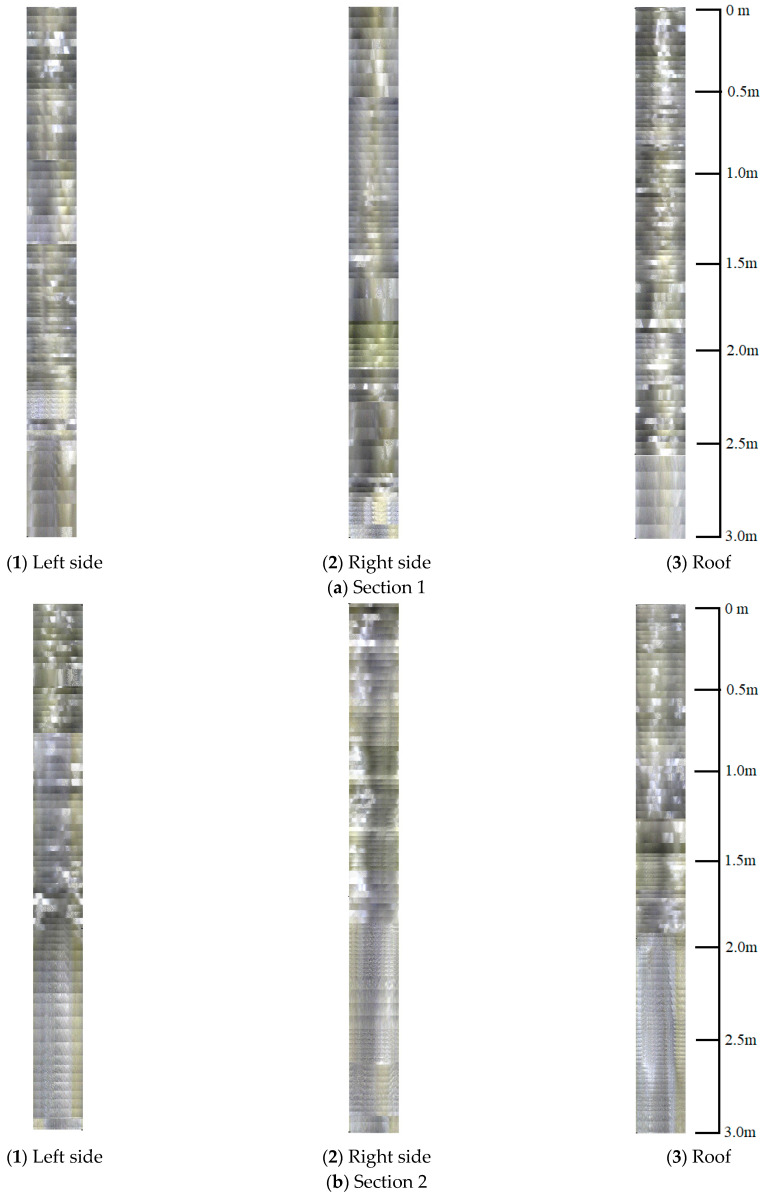
Expend view of peephole wall.

Expanding the image, as shown in Figure 13, provides a more intuitive view of the extent of loose rock damage at different borehole viewing positions. Overall, the rock fractures near the borehole entrance are more developed, with a prevalence of opening and wide opening fractures, indicating severe rock damage. In the middle section of the borehole, fractures are mainly open and slightly open. However, in the deeper borehole bottom section, fractures are not well developed.

In Figure 12a, the screenshot image shows that in Section 1, the borehole formation between 0.5 m and 1.5 m is irregular and not perfectly circular. Additionally, the high water content in this area leads to poor image clarity. During the inspection, there were multiple instances of the lens being obstructed by fractured surrounding rock, making the viewing process challenging. At the 2 m, 2.5 m, and 3 m positions, the borehole formation improves, and the integrity of the surrounding rock is better than in the shallower sections. At the 3 m depth, you can clearly see marks left by the rotating drill bit, indicating higher rock strength in this area. Based on the comprehensive viewing images in Figure 13a(1), the loose rock zone in this location ranges from 1.5 m to 2 m.

In Figure 12b, the right side of Section 1 is similar to the left side. The borehole formation from the borehole entrance to a depth of 1.5 m shows irregularities, fractured surrounding rock, significant deformation, and a high water content. The viewing quality improves at the 2 m depth position, and it is excellent at the 3 m depth position. Based on the comprehensive viewing images in Figure 13a(2), the loose rock zone on the right side of Section 1 ranges from 1.5 m to 2 m.

In Figure 12c, the top section of Section 1 has poorer viewing quality than the left and right sides. There are several reasons for this: the borehole formation is irregular, the lens is obscured by water mist, and the soft surrounding rock causes the area around the borehole to be more fractured. In the three images at 0.5 m, 1 m, and 1.5 m, there are noticeable broken and fallen particles, and the borehole edges are not very smooth. Although there is some improvement in the surrounding rock at the 2 m and 2.5 m positions compared to the first 1.5 m, the borehole shapes are still irregular, and there is significant surrounding rock deformation. At the 3 m depth position, the borehole formation is generally circular, and the surrounding rock here has almost no deformation. From the comprehensive viewing images in Figure 13a(3), it is clear that the loose rock zone at the top of Section 1 ranges from 1.5 m to 2 m.

Compared to Section 1, the viewing quality of the borehole images in Section 2 has significantly improved. The deeper the borehole, the higher the rock strength, and the more pronounced the thread-like marks left by the rotating drill bit. In some cases, you can even clearly see the triangular marks left by the drill bit on the borehole bottom. In Figure 12d, the left side of Section 2 has a concentration of fractured rock around the borehole entrance to a depth of 1 m. In this area, the borehole formation is irregular, and there are well-developed fractures with high water content. However, in deeper positions, specifically from 1.5 m to the borehole bottom, the surrounding rock structure is better, and no significant fractured areas are observed. Based on the comprehensive viewing images in Figure 13b(1), the loose rock zone in the left side of Section 2 ranges from 1 m to 1.5 m.

In Figure 12e, the right side of Section 2 is similar to the left side. The deeper the probe goes, the better the viewing quality becomes. The fractured rock zone is concentrated around the borehole entrance to a depth of 1 m. Irregular borehole formations and well-developed fractures are observed in this area. However, between the 1.5 m position and the borehole bottom, the surrounding rock structure is better. Based on the comprehensive viewing images in Figure 13b(2), the loose rock zone in the right side of Section 2 ranges from 1 m to 1.5 m.

In Figure 12f, the top section of Section 2 shows significant improvement in viewing quality compared to the top section of Section 1. The closer the probe is to the deeper part of the surrounding rock, the greater the improvement. The fractured rock zone, similar to the shoulders, is mainly concentrated around the borehole entrance to a depth of 1.5 m. However, between the 2 m position and the borehole bottom, the surrounding rock structure and integrity are excellent. No bedding or fractures are observed in this area. Based on the comprehensive viewing images in Figure 13b(3), the loose rock zone in the top section of Section 2 ranges from 1 m to 1.5 m.

These results are consistent with the identification of loose rock zones using self-sensing optical fiber anchors. This study demonstrates that self-sensing optical fiber anchors can not only monitor axial forces but also assess the degree of surrounding rock fragmentation and the extent of loose zones.

## 5. Intelligent Monitoring System Development

How to collect monitoring data and conduct rapid analysis is the key to determining whether “intelligent” monitoring can be realized. Therefore, a set of intelligent monitoring systems should be developed to combine each independent fiber optic bolt into a comprehensive and three-dimensional monitoring system, so as to realize comprehensive monitoring of different positions and depths of the tunnel. It provides a reliable data source for analyzing the stress on bolts at different depths and the thickness of the loose ring at different positions in the surrounding rock and provides an auxiliary decision factor for engineers and technicians to adjust supporting parameters and optimize construction technology.

**Figure 14:**
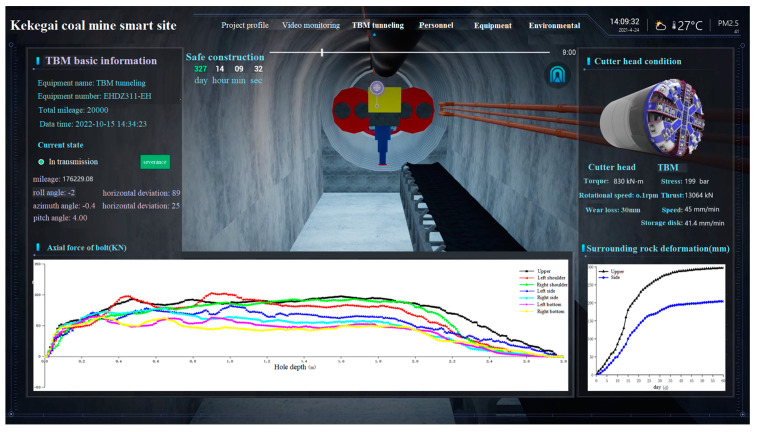
Intelligent monitoring system.

The current wireless transmission network is used to import monitoring data into the monitoring system, as shown in Figure 14, and the monitoring results can be seen in real time.

## 6. Conclusions

This article proposes a new self-sensing optical fiber anchor, made by improving the stirrer, to monitor the axial force on the bolt and the loose zone of surrounding rock. It can discover the rules of anchor axial forces and the development of loosening zones throughout the tunnel’s entire lifecycle. This new self-sensing fiber optic anchor has been successfully applied in an open TBM tunneling project in an inclined shaft in the Kekegai coal mine, and the main conclusions are drawn as follows:

(1) A new self-sensing fiber anchor was designed and made by improving the stirrer. The stirrer’s length was increased from 18 cm to 30 cm, and it was combined with fiber optic technology, which is conducive to large-scale field application due to its easy installation and cheap price.
(2) The monitoring results show that the axial force curve can be divided into four segments: the borehole area, the loosening zone, the stable zone, and the anchoring zone. The axial force exhibits an overall trend of rapid increase, intense fluctuations, gradual stabilization, and slow decline. This indicates that as the depth increases, the influence of excavation on the surrounding rock gradually decreases and the stress approaches its original state.
(3) The monitoring results show that the loosening zone ranges from 1 m to 1.7 m, and the anchoring zone ranges from 0.3 m to 1.5 m, indicating that the local position needs to strengthen the support and increase the amount of cartridge.
(4) The results of bolt dynamometers and borehole inspections verify the accuracy of the self-sensing fiber optic bolt in axial force monitoring and loose circle identification, ensuring the reliability and economy of engineering applications. On this basis, the intelligent monitoring system can realize the real-time non-destructive monitoring of the whole life cycle of tunnel construction and provide a basis for timely adjustments to the parameters of TBM tunneling and support.

## Data Availability

Data are contained within the article.
